# Verification of flow velocity measurements using micrometer-order thermometers

**DOI:** 10.1038/s41598-021-02877-w

**Published:** 2021-12-10

**Authors:** Naoki Takegawa, Masahiro Ishibashi, Aya Iwai, Noriyuki Furuichi, Toshihiro Morioka

**Affiliations:** 1 National Metrology Institute of Japan (NMIJ), National Institute of Advanced Industrial Science and Technology (AIST), 1-1-1, Umezono, Tsukuba, Ibaraki 305-8563 Japan; 2 Japan Gasmeter Industry Association, 1-8-13, Toranomon, Minato, Tokyo, 105-0001 Japan

**Keywords:** Physics, Fluid dynamics

## Abstract

In flow velocity measurements, resolution, miniaturization, and accuracy of measuring devices are important issues because the measuring devices significantly affect the flow in the micro-space, sonic flow, and turbulent flow. We studied recovery temperature anemometry (RTA) using micrometer-order thermometers and evaluated its validity in two velocity ranges (40–90 and 315–420 m/s) by conducting two experiments and a numerical simulation. The results confirmed that the difference between the reference velocity and RTA was within 5% in the velocity range 60–90 m/s for both the thermocouple and platinum thermometer given the same recovery temperature coefficient of 0.83. It is a valuable finding that velocity measurement by RTA is independent of the type of thermometer used. This suggests that the accuracy of about 5% can be guaranteed even without calibration by giving the recovery temperature coefficient according to the thermometer geometry, which is an excellent advantage not found in other anemometers. Furthermore, the supersonic flow measured using RTA agrees well with the simulation results and theoretical trends. Our findings ensure that the micrometer-order point measurement of flow velocity, which is difficult with existing anemometers, using RTA is possible over a wide range of flow velocities.

## Introduction

Airflow measurement is extremely important for clarifying natural phenomena and for engineering applications. In the industrial field, there is a need for the measurement of a wide range of flow velocities (from low-speed flow in a clean room to supersonic flow in a rocket engine nozzle). Furthermore, there has been a high demand for flow velocity measurements in a minute space (approximately micrometers to millimeters) due to miniaturization of manufacturing in recent years. To this end, a variety of flow velocity measurement methods have already been proposed, such as pitot tubes^1^, hot wire anemometry^2–4^, particle image velocimetry (PIV)^5–7^, and laser Doppler velocimetry (LDV)^8,9^. Although hot wire anemometers are widely used for turbulence measurements, two or more probes supporting the thin wire often affect flow, and the insertion angle of the probes is an issue that needs to be considered^10,11^. Furthermore, the measured flow velocity is a line average (line measurement) because the hot wire length ranges from several hundred micrometers to several millimeters in generally. Optical methods such as PIV and LDV are more expensive than pitot tubes and hot wire anemometers, thereby hindering their introduction. Additionally, it is difficult to apply these methods to practical situations, such as flow velocity measurement in pipes in plants and for outdoor flow measurement because of the need to insert tracer particles and laser beams.

**Figure 1:**
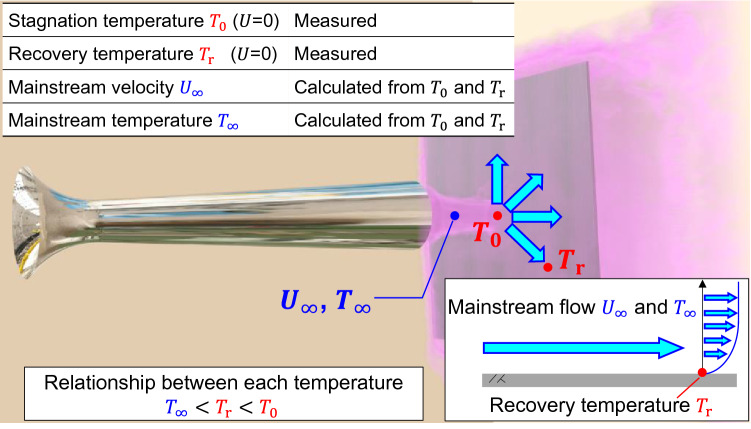
Conceptual diagram of stagnation temperature *T*_0_ and recovery temperature *T*_r_. Both *T*_0_ and *T*_r_ are points on the wall, and therefore, the flow velocity is zero. However, *T*_r_ is lower than *T*_0_ because of the effect of the temperature gradient in the main flow. The flow velocity in the mainstream is proportional to the square of the difference between *T*_0_ and *T*_r_.

Ishibashi^12,13^ proposed recovery temperature anemometry (RTA), which is based on the difference between the recovery temperature and stagnation temperature. Figure 1 illustrates a jet impacting a wall and then flowing over the wall surface. In a gas flow with a Prandtl number (kinematic viscosity/thermal diffusivity) less than 1.0, the wall temperature of an object that does not block the flow is smaller than the stagnation temperature *T*_0_ due to the large effect of thermal diffusion. This temperature is called the recovery temperature *T*_r_.

1 $${T_0} = {T_\infty } + \frac{\gamma - 1}{2}{M_\infty }^2{T_\infty } = {T_\infty } + \frac{{{U_\infty }^2}}{{2{C_p}}}$$

2 $${T_\text{r}} = {T_\infty } + r\frac{\gamma - 1}{2}{M_\infty }^2{T_\infty } = {T_\infty } + r\frac{{{U_\infty }^2}}{{2{C_p}}}$$

where *T*_∞_, *M*_∞_, *U*_∞_, *γ*, *C*_*p*_, *r* denote the mainstream temperature, mainstream Mach number, mainstream flow velocity, specific heat ratio, specific heat of constant pressure, and recovery temperature coefficient respectively. It is possible to measure the velocity distribution based on the temperature field because the temperature difference between *T*_0_ and *T*_r_ is proportional to the square of the main flow velocity.

3 $${U_\infty } = \sqrt{\frac{2{C_p}\left( {T_0} - {T_\text{r}} \right)}{1 - r}}$$

It is possible to realize a micrometer-order point measurement of flow velocity using a small temperature sensor to measure *T*_r_, which is difficult with existing anemometers. Ishibashi^12,13^ measured the flow in a sonic nozzle using thermocouples and captured the shock waves generated in the nozzle. However, the accuracy of RTA and the applicability of other thermometers are yet to be verified. Schmirler^14^ proposed new RTA that calculates the flow velocity from two recovery temperature coefficients of two thermometers located close to each other. However, the validated flow velocity range is limited to 120–260 m/s, and only one type of thermometer with a relatively large sensor diameter of 1.5 mm was used.

**Figure 2:**
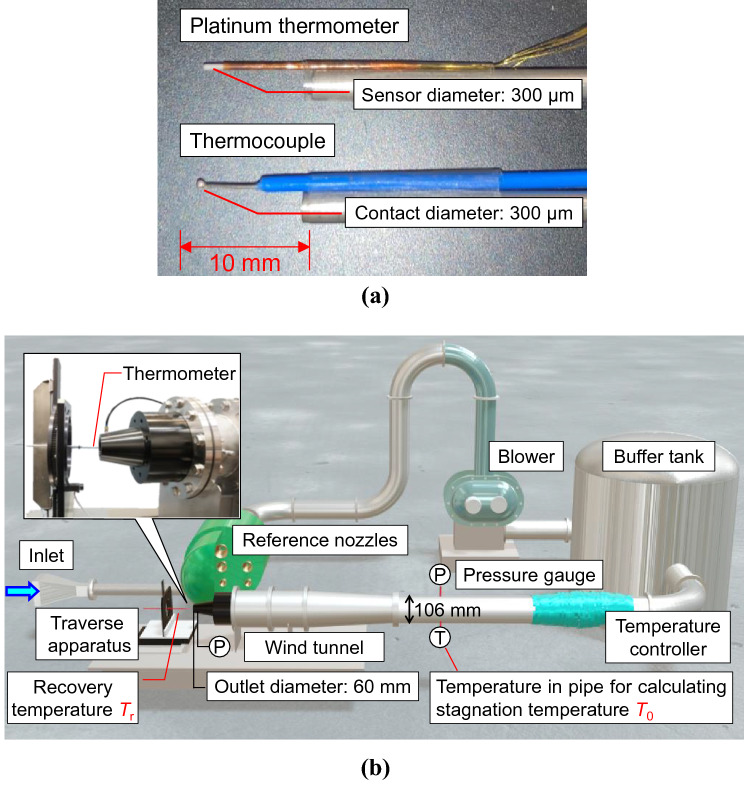
Conceptual diagram of the comparison experiment with reference velocity. (**a**) Thermometers for measuring the recovery temperature. The contact diameter of the thermocouple is approximately 300 μm. The sensor diameter of the platinum thermometer is approximately 300 μm, and sensor length is approximately 1.5 mm. (**b**) Conceptual diagram of test section. The reference velocity was derived by dividing the flow rate of the reference nozzles installed upstream of the test section using the cross-sectional area of the wind tunnel outlet. The reference nozzles can generate a constant flow rate because the flow velocity at the throat with the smallest cross-sectional area becomes equal to the speed of sound when a differential pressure of a certain level or more is applied by the blower. The thermocouple or platinum thermometer used to measure the flow velocity (recovery temperature) is attached to the traverse apparatus at the downstream side of the wind tunnel; the measured value by RTA is compared with the value of the reference velocity.

In this study, the validity of RTA was verified in two velocity ranges (40–90 and 315–420 m/s) through two experiments and a numerical simulation. In the velocity ranges, the accuracy of RTA has not been verified so far. Furthermore, two types of probe temperature sensors were verified to extend the applicability of RTA. A thermocouple (contact diameter: 300 μm) and platinum thermometer (sensor diameter: 300 μm) shown in Fig. 2a were used to measure the recovery temperature. The two probe temperature sensors were selected to be as small as possible, and the same value of recovery temperature coefficient *r* of 0.83 was used for both sensors. The accuracy of RTA using these thermometers was evaluated using the reference velocity (range: approximately 40–90 m/s) with an expanded uncertainty of 0.63% (coverage factor: 2)^15^ owned by the National Metrology Institute of Japan. In addition, RTA was applied to the measurement of flow velocity in a small sonic nozzle with a throat diameter of 13.4 mm; the accuracy of the measurement in sonic flow was evaluated by comparing it with the results of the 3D numerical simulations. Therefore, we evaluated the validity of RTA from various viewpoints, such as comparison experiments with the national standard, theory, and numerical simulation.

The results of this study confirmed that the difference between RTA and the reference velocity was within 5% in the velocity range of 60–90 m/s, regardless of the type of thermometer. This suggests that the accuracy of about 5% can be guaranteed even without calibration by giving the recovery temperature coefficient according to the thermometer geometry, which is an excellent advantage not found in other anemometers. These results and the presentation of the benefits have not been described in other papers. Furthermore, the sonic flow in the nozzle measured by RTA was in good agreement with the simulation results and theoretical trends. Various existing inexpensive thermometers have enabled the micrometer-order point measurement of flow velocities over a wide range of flow velocities. It is confirmed in this study that RTA can be used in point velocity measurement, which was earlier difficult with existing anemometers, over a wide range of flow velocities.

## Results

### Comparison experiment with reference velocity

A comparison experiment with a national standard of flow velocity was conducted to evaluate the measurement accuracy of RTA. The reference velocity^15^ was derived from a flow rate reference^16^ based on reference nozzles. As depicted in Fig. 2b, the reference nozzles are installed in parallel at the upstream of the test section, and a flow rate of 5–1000 m^3^/h can be generated in a stable manner by applying the critical back pressure ratio using the blower^16^. The reference velocity at the sensor position fixed to the traverse apparatus is calculated from the volumetric flow rate at the wind tunnel outlet, cross-sectional area (outlet diameter: 60 mm), and boundary layer thickness. The derivation of the reference flow velocity is explained in detail by Iwai^15^. When calculating the flow velocity using RTA, both *T*_r_ and *T*_0_ are required, and the *T*_0_ of the fluid on the streamline is measured using a platinum thermometer on the upstream side of the wind tunnel. *r* is necessary to calculate the flow velocity, and it is known to be approximately 0.88^17,18^ for parallel plates and 0.825–0.875^19,20^ for cone and parabolic bodies in a laminar boundary layer. In this study, an *r* of 0.83 was used for both the thermocouple and platinum thermometers to match the experimental results with the reference velocity. The difference observed between *r* = 0.83 and the literature value of *r* = 0.825–0.875 is attributed to the shape of the thermometers being a probe type. RTA is a physical model, and thus, does not require many coefficients; however, *r* needs to be provided as a thermometer-specific value.

### Accuracy of flow velocity measurement by RTA

**Figure 3:**
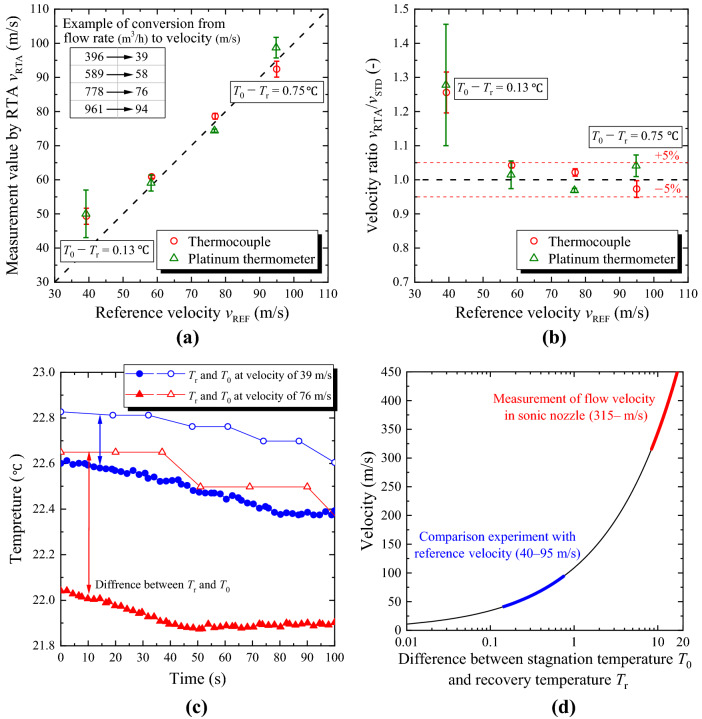
Experimental results comparing RTA with reference velocity. Experiments were performed three times under the same conditions. The plots show the mean values, and the error bars show the standard errors. (**a**) Comparison between *v*_REF_ and *v*_RTA_ based on absolute values. The upper left part of the graph shows an example of the conversion from the flow rate to the standard velocity in this experiment. The difference between *T*_0_ and *T*_r_ is provided for reference under the conditions of flow velocities of approximately 40 m/s and 90 m/s. (**b**) Comparison between *v*_REF_ and *v*_RTA_ based on ratios. The red dashed line indicates the difference of ± 5% from the reference velocity. (**c**) Time variation of *T*_0_ and *T*_r_ measured using platinum thermometers at flow velocity of 39 and 76 m/s. (**d**) Relationship between the temperature difference between *T*_0_ and *T*_r_ and the flow velocity. The specific heat of constant pressure *C*_*p*_ and the recovery temperature coefficient *r* are set to 1006 (J kg^−1^ K^−1^) and 0.83, respectively. The relationship was calculated using Eq. (3). The velocity ranges of the two experiments conducted in this study are also presented.

Figure 3a,b illustrate the relationship between the reference velocity *v*_REF_ and the value measured by RTA *v*_RTA_ based on absolute values and ratios, respectively. Figure 3b shows that the maximum difference between *v*_REF_ and *v*_RTA_ is approximately 30% when the flow velocity is approximately 40 m/s. The standard error of *v*_RTA_ is also larger than that of the other velocity points. It is considered that the accuracy of temperature measurement affects the measurement accuracy of RTA, and the difference between *T*_0_ and *T*_r_ is as small as approximately 0.13 °C at approximately 40 m/s. In the velocity range of 60–90 m/s, the temperature difference between *T*_0_ and *T*_r_ increases to approximately 0.75 °C, and the difference between *v*_REF_ and *v*_RTA_ is within 5% for both the thermocouple and platinum thermometer. Therefore, in the range of large flow velocities where the temperature difference between *T*_0_ and *T*_r_ increases, a high accuracy can be expected regardless of the type of thermometers. The standard error of *v*_RTA_ for both the thermocouple and the platinum thermometer increased for *v*_REF_ = 90 m/s compared to that for *v*_REF_ = 75 m/s. This can be attributed to the increase in temperature fluctuation in the test section when the maximum flow rate of 1000 m^3^/h is generated using the reference nozzles and blower. Figure 3c shows the time variation of *T*_0_ and *T*_r_ measured using platinum thermometers, and Fig. 3d depicts the relationship between the temperature difference between *T*_0_ and *T*_r_ and the flow velocity calculated using Eq. (3).

### Velocity measurement and numerical simulation of flow in a sonic nozzle

**Figure 4:**
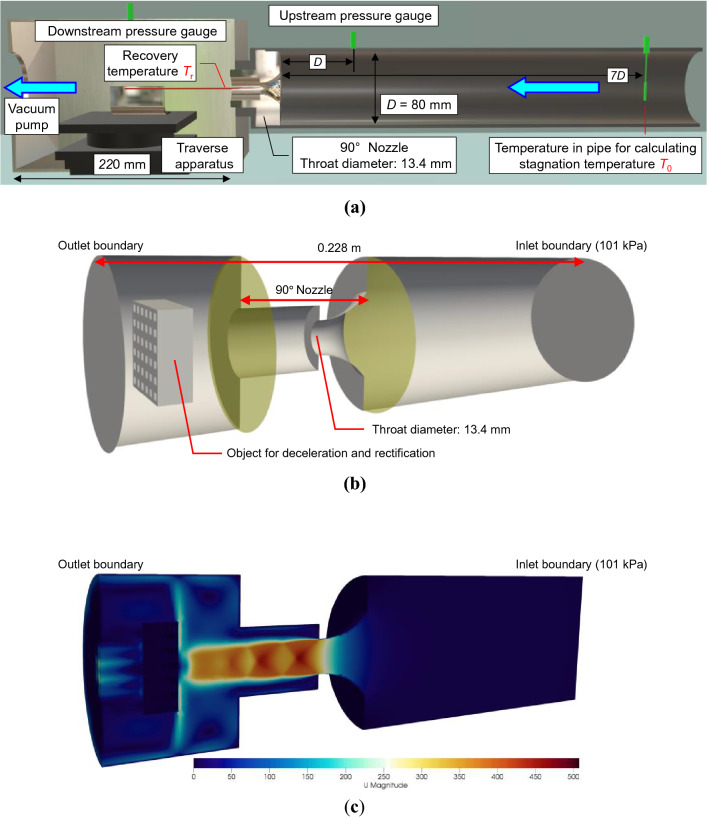
Overview of the experiment and numerical simulation of flow in a sonic nozzle. (**a**) Conceptual diagram of experimental apparatus. A 90° nozzle without a diffuser was installed upstream of the traverse apparatus. *T*_r_ was measured from the downstream of the nozzle using the thermocouple or platinum thermometer attached to the traverse apparatus. *T*_0_ was measured in the region where the flow velocity upstream of the nozzle was sufficiently low. The pressure gauges installed upstream and downstream of the nozzle were used to check the approximate back pressure ratio. (**b**) Simulation region. The space between the yellow-colored regions represents the 90° nozzle, and the nozzle shape is reproduced as in the experiment. (**c**) Velocity contour at 0.005 s after the start of simulation. Shock wave generation is observed downstream of the nozzle throat.

After verifying the validity of the flow velocity measurement by RTA through comparison with the reference velocity, RTA using the thermocouple or platinum thermometer was applied to the flow velocity measurement inside a sonic nozzle. Unlike the experiment with the reference velocity described above, there is no reference value at each spatial position inside the nozzle, and therefore, the experimental results are compared with the results of the numerical simulations. Figure 4a presents an overview of the experiment. A 90° sonic nozzle (throat diameter: 13.4 mm) without a diffuser is installed upstream of the traverse apparatus, and the critical back pressure ratio of the nozzle is approximately 0.53. A vacuum pump and control valve were used to provide sufficient and constant differential pressures to the nozzle. The approximate back pressure ratio during the experiments was obtained from pressure gauges installed upstream and downstream of the nozzle. The *T*_*r*_ was measured using a thermocouple or platinum thermometer fixed to the traverse apparatus, and the *T*_0_ of the fluid in the streamline was measured using a platinum thermometer on the upstream side of the nozzle. The following three velocities were verified: (1) flow velocity at the center of the throat, (2) flow velocity in the axial direction, and (3) flow velocity in the radial direction 1 mm downstream of the throat.

Numerical simulations were performed using OpenFOAM (Open source Field Operation And Manipulation)^21^, which is a fluid analysis software, to perform unsteady three-dimensional simulations. For the simulation region shown in Fig. 4b, the shape inside the 90° nozzle was reproduced with the same dimensions as in the experiment. However, the spaces upstream and downstream of the nozzle did not have the same shape and volume as in the experiment. Therefore, a rectifying section was installed downstream of the nozzle to equalize the flow velocity at the outlet boundary and decrease the simulation area. In the simulations, the inlet boundary and internal pressure of the nozzle were set to 101 kPa, and a differential pressure greater than the critical back pressure ratio was applied to the outlet boundary as in the experiment. The mass flow rate at the throat after 0.005 s was stable, and the flow field after 0.005 s was provided as the calculation result. Figure 4c shows the velocity contours as an example of the calculation results.

### Comparison with the theoretical value at the center of the nozzle throat

**Figure 5:**
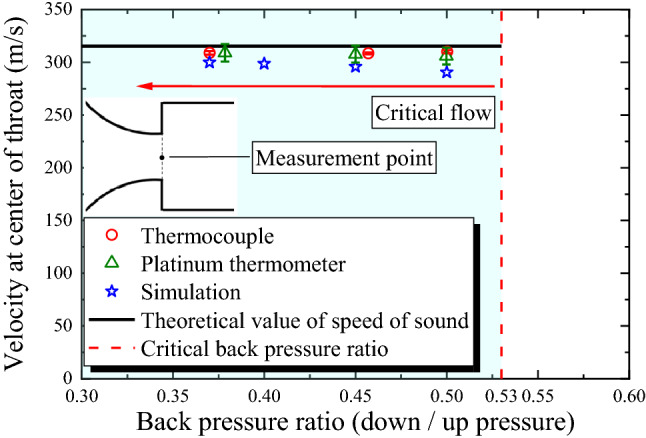
Comparison of RTA and simulation results with theoretical values for flow velocity at the center of the nozzle throat. The experiments were performed three times under the same conditions. The plots show the mean values and the error bars show the standard errors. The critical back pressure ratio of the 90° sonic nozzle is approximately 0.53.

Figure 5 depicts the comparison of the results of RTA and numerical simulations at each back pressure ratio with the theoretical values for the flow velocity at the center of the nozzle throat. The theoretical value of the speed of sound *c* is derived from the following equation.

4 $$c = \sqrt {\gamma R{T_{\text {th}}}} = \sqrt {\gamma R\left( {\frac{2}{\gamma + 1}{T_0}} \right)}$$

where *R* denotes the gas constant (J·K^−1^·kg^−1^), and *T*_th_ represents the temperature at the nozzle throat. The theoretical value of the speed of sound calculated using this equation is approximately 315 m/s. For each back pressure ratio (0.5, 0.45, 0.40 and 0.37) less than the critical back pressure ratio of 0.53, the flow velocity at the throat measured by RTA is approximately constant despite the different pressure differences. This effectively captures the qualitative characteristics of the sonic nozzle. The velocities obtained by RTA and numerical simulation are up to approximately 8% lower than the theoretical value of 315 m/s because the theoretical equation for the speed of sound does not consider the effect of the curvature of the nozzle inlet which reduces the flow velocity at the center of the throat^22–26^. RTA and numerical simulations show a trend in harmony with the previous studies^22–26^. Considering the back pressure ratio of 0.45 as an example, the flow velocities measured by RTA and numerical simulations agree within a range of approximately 3%, which indicate that the measurement accuracy is high. For reference, when the back pressure ratio is less than 0.5, the difference between *T*_0_ and *T*_r_ measured at the center of the throat was approximately 8 °C or more. These results confirm that RTA can be applied to sonic flow in small-diameter nozzles because low-disturbance measurement can be achieved using a temperature sensor on the order of micrometers.

### Traverse measurement in axial and radial directions

**Figure 6:**
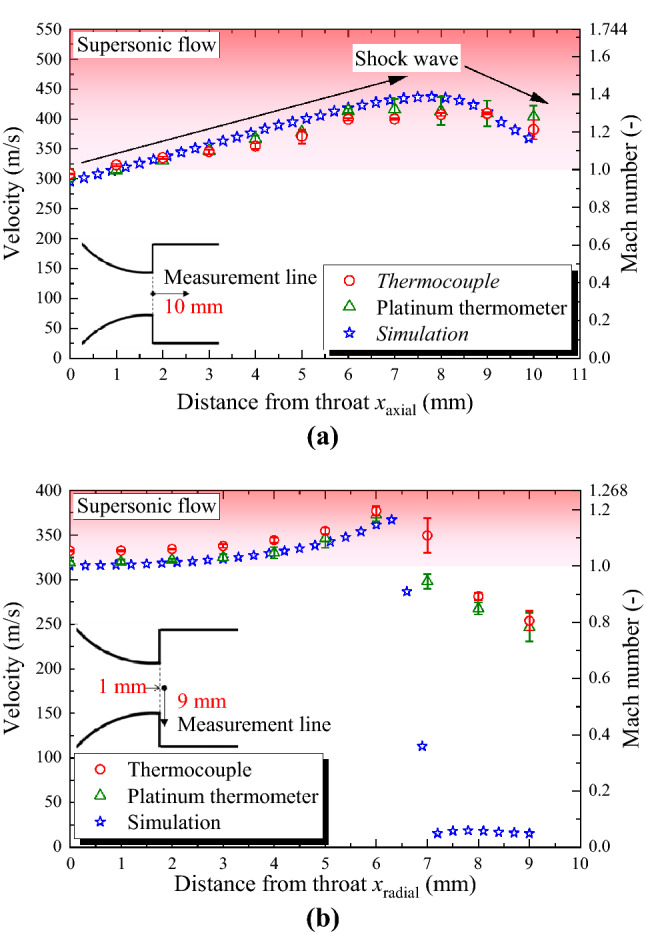
Traverse measurement of flow velocity in a sonic nozzle in the axial and radial directions. The experiments were performed three times under the same conditions. The plots show the mean values and the error bars show the standard errors. The experimental and simulation results were obtained at a back pressure ratio of 0.4. (**a**) Axial velocity distribution from the center of the throat to 10 mm downstream. (**c**) Radial velocity distribution at 1 mm downstream of the throat. The throat radius of the nozzle is approximately 6.7 mm.

Figure 6 depicts the traverse measurement of flow velocity in the sonic nozzle in the axial and radial directions. Figure 6a shows the results of the traverse measurement from the center position of the throat to *x*_axial_ = 10 mm at 1 mm intervals in the downstream axial direction. Each result is obtained under the condition that the back pressure ratio is fixed at 0.4. In RTA and the numerical simulation, a supersonic flow is confirmed downstream of the throat, and in Fig. 4c, an oblique shock wave is generated downstream of the throat in the simulation. In RTA, the flow velocity and Mach number increase to approximately 420 m/s and 1.3, respectively, at *x*_axial_ = 7 mm, which indicates that RTA is valid for supersonic flow. The flow velocity decreases after *x*_axial_ = 7 mm, and the shock wave phenomenon in the nozzle can be captured. Although experiments and numerical simulation show good agreement in terms of flow velocity at the center of the throat, a difference of up to 10% is observed downstream of the nozzle throat. This is attributed to the fact that the numerical simulation does not fully reproduce the space inside the traverse apparatus downstream of the nozzle. Although the pressure ratio between the inflow and outflow boundaries was set to 0.4, the pressure field at the nozzle exit in the simulation may be different from that in the experiment, and this effect is expected to occur downstream of the nozzle throat.

Figure 6b depicts the results of the radial traverse measurements at 1 mm downstream of the throat. The measurement points were set at intervals of 1 mm at *x*_radial_ = 0–9 mm, and *x*_radial_ = 0 represents the center of the nozzle throat. Each result was obtained under the constant back pressure ratio of 0.4. At *x*_radial_ = 0–6 mm, the flow velocity increases as *x*_radial_ increases because of the influence of the inlet curvature described above^22–26^, and RTA, and the simulation results show extremely good agreement. The throat radius of the 90° nozzle is 6.7 mm, and it is presumed that the region of *x*_radial_ = 7–9 mm is outside the jet of supersonic flow; therefore, a decrease in flow velocity is confirmed. However, there is a large difference between RTA and the numerical simulation in terms of the reduction rate of flow velocity in the region outside the jet, and the experimental results indicate that the flow velocity is approximately 250 m/s even in the region considered to be outside the jet. Normally, *T*_0_ and *T*_r_ at the same point have the same value when the flow velocity is close to zero. *T*_0_ and *T*_r_ at the same point are required to accurately measure the flow velocity outside the jet, and in this experiment, *T*_0_ in the streamline was calculated from the temperature measured upstream of the nozzle. Therefore, *T*_r_ outside the jet (outside the streamline) did not match *T*_0_, even when the flow velocity was near zero. Low-disturbance measurements of *T*_0_ and *T*_r_ at the same point will be considered in the future.

## Discussion

In this study, a velocity measurement method (RTA) based on *T*_r_ and *T*_0_ was verified. The validity of RTA based on the flow velocity measurement traceable to the national standard was uniquely verified. When RTA was applied to the flow in the sonic nozzle, the experimental and simulation results were in good agreement, which suggest the possibility of measuring the supersonic flow using RTA. Furthermore, similar results were confirmed with a thermocouple and platinum thermometer given the same recovery temperature coefficient, and it was found that the flow velocity measurement by RTA does not depend on the type of thermometer. These results indicate the possibility of realizing flow velocity measurement without calibration using Eq. (3) by giving a recovery temperature coefficient suitable for the thermometer shape (e.g., 0.83 for a probe sensor). The main feature of RTA is the simplicity and ease of micrometer-order point measurement using existing inexpensive thermometers. In comparison with existing anemometers, for hot wire anemometry, prior calibration is always performed. In addition, the hot wire anemometry requires a wire length of more than 100 times the wire diameter for achieving a uniform temperature distribution at the hot wire center, and the output results are line averaged rather than point. Optical methods such as PIV and LDV are not inexpensive and easy to use due to the bottleneck of using expensive equipment and lasers. The thermometers used in RTA are commercial products, and as mentioned above, RTA can measure the flow velocity without calibration by using the appropriate recovery temperature coefficient. Therefore, RTA can be easily used by engineers.

The issues of RTA at this stage are described below. In the region of small flow velocity, the difference between *T*_0_ and *T*_r_ is also small, which may make increase the difficulty of application of RTA in terms of the accuracy of temperature measurement. In this study, the difference between *T*_0_ and *T*_r_ is approximately 0.13 °C at approximately 40 m/s. To expand the application range and improve the measurement accuracy of RTA, measurements of *T*_0_ and *T*_r_ at the same point are necessary. As a condition for the application of RTA, the Prandtl number of the fluid must be less than 1.0 in terms of the difference between *T*_0_ and *T*_r_, for instance, RTA cannot be applied to water at 20 °C. (Prandtl number: approximately 7.0).

Several prospects for measurements using RTA exist in the future. For instance, the measurement range of thermocouples extends to high temperatures, and therefore, RTA is applicable to high-enthalpy flow, characteristic of the aerospace field. Moreover, the heat capacity of small temperature sensors is small, and therefore, RTA is expected to demonstrate a high response to velocity fluctuations and can be applied to turbulence research.

## Methods

### Experimental procedures and equipment

In the comparison experiment with the reference velocity and the velocity measurement experiment in the sonic nozzle, the flow was generated by sucking air with a relative humidity of less than 50% from the atmosphere. The thermocouple measurement system comprised a sensor (TPK-01, Mother tool) and a logger (TM-947SD, Mother tool). The platinum thermometer measurement system also comprised a sensor (special order, Netsushin) and a logger (Multimeter 2001, Keithley). *T*_0_ and *T*_r_ are measured at intervals of about 10 and 1 s, respectively, and are the average values obtained over 100 s of measurement. *T*_0_ was derived using

5 $${T_0} = \left( 1 + \frac{\gamma - 1}{2}{M_{\text{p}}^2} \right){T_{\text{p}}}$$

where *M*_p_ denotes the Mach number in the pipe, and *T*_p_ denotes the temperature in the pipe. If the flow velocity through the pipe is sufficiently low, the measured temperature can be treated as *T*_0_. For the measurement accuracy of each system in the comparison experiment with the reference velocity, all reference nozzles were calibrated with a constant volume tank, which is the national standard for the gas flow rate; furthermore, the expanded uncertainty of the calibration is approximately 0.17% (coverage factor: 2)^27^. The flow rate fluctuation of 5–1000 m^3^/h generated by the reference nozzles and blower is controlled to ± 0.1%/10 min by the buffer tank and temperature controller. The expanded uncertainty of the flow rate is approximately 0.28% (coverage factor: 2)^28^. The expanded uncertainty of the reference velocity based on the flow rate reference is estimated to be approximately 0.63% (coverage factor: 2)^15^. The traverse apparatus (Siguma Koki) shown in Fig. 4a has a movement accuracy of 3 μm and can be operated by an actuator from outside the traverse apparatus.

### Numerical simulation

Three-dimensional unsteady numerical simulations of a compressible fluid with an ideal gas were performed using OpenFOAM to verify the flow velocity inside the nozzle measured by RTA. The finite volume method was used to solve the governing equations (continuity, momentum, and energy equations) given by

6 $$\frac{\partial \rho }{{\partial t}} + \nabla \cdot \left( {\rho \varvec u} \right) = 0$$

7 $$\frac{\partial \rho {{\varvec u}}}{{\partial t}} + \nabla \cdot \left( {\rho {\varvec u \varvec u}} \right) = - \nabla p + \nabla \cdot \left[ {\mu \left\{ {\nabla {\varvec{u}} + {{\left( {\nabla {\varvec{u}}} \right)}^T}} \right\}} \right] - \nabla \left( { - \frac{2}{3}\mu \nabla \cdot {\varvec{u}}} \right)$$

8 $$\frac{\partial \rho e}{{\partial t}} + \nabla \cdot \left( {\rho e {\varvec{u}}} \right) + \frac{\partial \rho K}{{\partial t}}+\nabla \cdot \left( {\rho K {\varvec{u}}} \right) = - \nabla \cdot \left( {p {\varvec{u}}} \right) + \nabla \cdot \left( {k\nabla T} \right)$$

where *ρ*, *t*, ***u***, *p*, *μ*, *E*, *k*, and *T* denote the density, time, velocity vector, pressure, viscosity coefficient, total energy per unit mass, thermal conductivity, and temperature, respectively. *μ* was calculated using Sutherland’s equation, and *k* was calculated from the viscosity coefficient and the constant volume specific heat. Values of 1005 and 717.9 (J·K^−1^·kg^−1^) were used for the constant pressure specific heat and constant volume specific heat, respectively. The pressure and velocity were coupled using the rhoPimpleFoam solver, which is a combination of PISO (pressure implicit with splitting operators)^29^ and SIMPLE (Semi-Implicit Method for Pressure)^30^. The *k–ω* shear stress transport^30^ was adopted as the turbulence model. A second-order accurate linear upwind difference, linearUpwind limited, was used as the advection term for the velocity vector ***u***, kinetic energy *K*, internal energy *e*, turbulent energy *k*, and energy dissipation rate *ω*. The total number of cells was approximately 1.69 million, and the mesh in the axial direction (x) was approximately 1.3 times longer than that in the radial direction (y, z). In the detailed mesh region inside the nozzle, the length in the radial directions was approximately 0.24 mm and was approximately 0.96 mm in other regions. Boundary layer meshes with a mesh height of about 0.2 times the normal mesh height were inserted on the wall surface for three layers. Ryzen Threadripper 3990X (Advanced Micro Devices) was used as the CPU, and the number of parallelisms was set to 118. A variable time width was set for the calculation, where the Courant number did not exceed 0.7. The conditions for the numerical simulations are listed in Table 1.

**Table 1:** Conditions of the numerical simulations.

Total number of cells	1,691,062
Solver	rhoPimpleFoam
Turbulence model	*k*–*ω* shear stress transport
Boundary condition for velocity	Inlet: zero gradient
	Nozzle wall: 0 m/s
	Outlet: InletOutlet (zero gradient with backflow prevention)
Boundary condition for pressure	Inlet: 101 kPa
	Nozzle wall: zero gradient
	Outlet: 50.5, 45.45, 40.4, or 35.35 kPa
Boundary condition for temperature	Inlet: 20 °C
	Nozzle wall: zero gradient
	Outlet: zero gradient
Courant number	0.7
Simulation time	0.005 s

## Data Availability

The data that support the findings of this study are available from the corresponding authors upon reasonable request.
